# Delineating the third age: joint models of older people's quality of life and attrition in Britain 2002–2010

**DOI:** 10.1080/13607863.2014.1003279

**Published:** 2015-02-02

**Authors:** Gindo Tampubolon

**Affiliations:** ^a^ Institute for Social Change, University of Manchester, Manchester, UK

**Keywords:** quality of life/well-being, mental health measures, epidemiology

## Abstract

**Objectives:** In the public mind, later life is being transformed by the emerging possibility of a flourishing third age with sustained quality of life. We draw trajectories of life quality measured using CASP-19 over eight years. We refine these trajectories by jointly modelling attrition, since older people tend to leave longitudinal studies (attrite) not at random.

**Methods:** Growth curve models are applied to the English Longitudinal Study of Ageing waves 1 to 5. Then joint model is estimated where attrition is considered. Extensive predictors are entered including demographic attributes, social and economic status, health conditions, and behaviours.

**Results:** Strong non-linear age trajectory of life quality is revealed by the growth curve models where the peak is achieved in the late 60s. Then the joint model uncovers the peak somewhat later in time, and also reveals secular improvement in life quality experienced by recent cohorts. Sharp estimates for many predictors of higher levels of life quality are also found.

**Conclusion:** For the first time, the trajectories of life quality in the third age are drawn and improvement across cohorts is demonstrated. The contributions are estimated for predictors amenable to intervention such as social capital. This can help in policy discussion on improving the lives of older people in the third age.

## Introduction

The importance and magnitude of the ageing population are increasingly being felt. The United Nations recently reported that at the turn of the century there were more people age 60 and over than age 5 and under (www.unfpa.org/public/home/publications/pid/11584, accessed 20 Feb. 2014) notes that at the turn of the century there were more people age 60 and over than aged 5 and under. And by 2050, there will be more older people than people age 15 and under. The ageing population demands concerted response worldwide as the increasing proportion of older people effects changed needs in health and social care. While much is known about functional limitations accompanying ageing, very little is known about the positive aspects of life quality as people age. Beyond conceiving the possibility of positive ageing, widely associated with the idea of the third age (Laslett, 1989), work must proceed to devise a construct of positive ageing that is susceptible to empirical investigation. But in both developed and developing countries few empirical studies have examined changes in older people's life quality over time especially using measure capable of cross-country comparison. The CASP-19 scale, standing for control, autonomy, self-realisation and pleasure, is a complex attempt at getting at older people's quality of life (Hyde, Wiggins, Higgs, & Blane, 2003).

When the life quality of older people is examined, generic population constructs of well-being such as happiness, life satisfaction and self-esteem are often used (Pinquart & Sörensen, 2000). It has been found that socio-economic status, social connections and everyday competence (primarily the absence of problems with activities of daily living) are important for maintaining a sense of being well in older ages. Although the generic, popular measures of life quality are helpful, their limitations in the context of older people's experience are variously known including weak theoretical foundation, inextricable links with their predictors and doubtful psychometric properties (Wiggins, Higgs, Hyde, & Blane, 2004). An alternative life quality measure that is specific to this age group was proposed and tested in the pages of this journal, i.e. CASP-19 (Hyde et al., 2003), and is now an integral part of the English Longitudinal Study of Ageing (ELSA) among other ageing studies. A few empirical investigations have used this construct to yield pictures of life quality, its predictors and social patterning in later life (Blane, Netuveli, & Montgomery, 2008; Howel, 2012; Netuveli, Wiggins, Hildon, Montgomery, & Blane, 2006; Wiggins et al., 2004; Wiggins, Netuveli, Hyde, Higgs, & Blane, 2008; Zaninotto, Falaschetti, & Sacker, 2009).

Both cross-sectional (Netuveli et al., 2006) and longitudinal studies (Zaninotto et al., 2009) show that quality of life linearly decreases with age, while the cross-sectional study additionally shows it changes non-linearly with age (squared). Thus whether trajectories of quality of life form a relentless march of decline or one with a peak is as yet undecided. Also unresolved is whether there is a difference in the levels of life quality between men and women once other social inequalities are considered, although there is evidence that women report higher life quality (Netuveli et al., 2006).

The CASP-19 construct is designed to be separate from its predictors in particular from wealth, social connections and health (Blane et al., 2008; Hyde et al., 2003;Wiggins et al., 2004). In the empirical literature, these are shown to affect quality of life in expected directions. Wealth or income variable was used in the cross-sectional study above and it is significant; it was also used in the longitudinal one but no result as to its magnitude and significance is reported. For instance, inadequate pension provision has been shown to correlate with a lower CASP-19 score (Wiggins et al., 2004).

Social connections in the forms of friendship and social support have also been examined, invariably finding people with higher quality of life tend to be more connected (Netuveli et al., 2006). In fact Zaninotto et al., (2009, p. 1301) concluded by suggesting that older people should ‘increase their network of friends and engage with the wider community’ while they can.

When included in the analysis, depression has a negative association with quality of life (Netuveli et al., 2006). In addition Blane et al. (2008) demonstrated with path analysis that depression is one of the routes through which health, specifically lung function limitation, affects quality of life. The authors also found direct associations between other functional limitations with quality of life.

As designed, CASP-19 has been demonstrated to be independently associated with health, net of other social--psychological factors (Wiggins et al., 2004). It has also been suggested that other health behaviour risks such as smoking and physical inactivities make up a separate predictor of CASP-19 (Blane et al., 2008, p. 2). Yet the empirical literature is still silent about them. They are explored here, including smoking, drinking and physical exercise.

In short, these different types of predictors, including demographic, social, psychological and physical health and behavioural predictors are examined sequentially, building upon earlier predictors in the list to draw complex trajectories of quality of life in older ages. But one other dimension, i.e. time, remains.

Because of its recent provenance, few have investigated how CASP-19 as a measure of life quality changes over time. Exceptional in this regard, Zaninotto and co-authors analysed life quality changes in the first three waves of ELSA by applying growth curve model. Importantly for longitudinal ageing studies, the analysis assumes missing at random attrition despite the study being ‘subject to attrition’ (p. 1304). To rectify this situation, a joint model of attrition and CASP-19 is used here, in particular shared parameter model (Wu & Carroll, 1988).

The paper aims to carefully draw the most recent trajectories of life quality in older people that are sensitive to their social patterning, and doing so while considering the inevitable attrition. In achieving this, clearer sense of the features of the third age will be apparent. For the first time the turning point in life quality will be presented and the contributions of an extensive set of predictors covering demographic, socio-economic, health and behavioural aspects will be assessed.

This work makes a number of contributions. First, by tracing non-linear change over a period of eight years, it presents and delineates the peak age at the centre of the third age. Second, it reveals cohort differences in levels of quality of life consistent with observed secular changes in cognitive functioning in recent cohorts or the ‘Flynn effect’ (Whalley, 2001, p. 56ff).

To focus the investigation, these questions are raised: how has the life quality of older people fared at the turn of the century? How can the trajectories of their experience be understood according to different socio-economic divisions in society? How much difference do health and behaviours additionally account for the trajectories? Do the recent cohorts show higher levels of CASP-19, indicating secular improvement?

The data and methods to answer these questions are briefly introduced in the next section. This is followed by a section where the results are presented; first, arising from the growth curve model that in application tends to assume away attrition; second, from the application of shared parameter model. The focus is on comparison between models where in one attrition is assumed random and in another it is deemed informative. Finally, the results are discussed by portraying robust and extensive trajectories of older people's life quality in recent times and relating them to broader social inequalities in healthy ageing.

## Methods and data

The English Longitudinal Study of Ageing (ELSA) is the primary longitudinal study of the English population aged 50 and over, designed to be closely comparable with its sister study, the US Health and Retirement Survey. A multidisciplinary study, ELSA collects data on participants' various health domains and social circumstances as they age. The original sample was drawn from the Health Survey for England in 1998, 1999 and 2001, known as wave 0. ELSA is downloadable from the UK Data Service (http://discover.ukdataservice.ac.uk/) as Study Number 5050.

Detailed information collected in waves 1 to 5 (2002–2010) were used including information on life quality, socio-economic status, social connections and social support, health conditions and health behaviours. Following Netuveli et al. (2006) and Zaninotto et al. (2009), the analytic sample starts in wave 1 with participants having complete CASP-19 items. The procedure of data collection in ELSA for both the main interviews and the nurse visits has been described elsewhere; see also www.ifs.org.uk/elsa. The University of Manchester's Research Ethics Committee approved the work. Quality of life is measured using CASP-19 scale which ranges from 0 to 57 and covers domains of control, autonomy, self-realisation and pleasure which together make up the acronym (Hyde et al., 2003). This measure focuses away from negative quality or illness. Because CASP-19 is designed to be free from its predictors such as health status, it is possible to examine the net contribution of each predictor in a complete model that includes demographic attributes, socioeconomic status, health conditions and risk behaviours.

These have, in fact, been variously examined in the empirical literature (Blane et al., 2008; Howel 2012; Netuveli et al., 2006; Wiggins et al., 2004, 2008; Zaninotto et al., 2009). Socio-economic status includes wealth quintiles (the poorest as reference), social class (routine class and the rest as reference, in order to focus on the routine versus non-routine cleavage; National Statistics Socio-Economic Classification, Rose & Pevalin 2003, p. 13), subjective social status elicited using Cantril ladder, education (degree level and less as reference), and marital status (comprising indicators for married/cohabiting, separated, widowed and single as reference). Social connections are constructed from responses about meeting friends, relatives and children (or speaking, or writing to them). Each of the responses ranges from 0 for never to 6 for three or more times a week; the sum gives social connections measure ranging from 0 to 18. Social support is constructed as a binary indicator using responses to whether, when facing serious problems, there is support from their spouses, children, relatives and friends some or a lot of the times. 

Health conditions include has a limiting longstanding illness, depression using continuous CES-D score, any cardiovascular problems comprising angina, hypertension, myocardial, congestive heart failure, heart murmur, arrhythmia, diabetes, stroke; any chronic obstructive pulmonary diseases (COPD) including bronchitis or asthma; any forms of cancer or malignant tumours; count of problems doing activities and instrumental activities of daily living comprising dressing, walking across a room, bathing, eating, getting in and out of bed, using the toilet, preparing a hot meal, shopping, making phone calls, taking medication, and managing money. Health behaviours included are smoking (or not) and physical exercise comprising vigorous, moderate and mild or less as reference.

The last set of predictors concerns time and sex. Cohort difference has not been examined in Britain and birth year is used to identify four birth cohorts: 1924--1930 (children of the Depression era), 1931–1941 (interwar babies), 1942–1947 (war babies), and 1948 onward (early baby boomers as reference). These cohorts are constructed to be comparable to the US Health and Retirement Study, a sister study of ELSA, facilitating future cross-country comparison. But there is no comparison possible with the American Oldest-old cohort since in ELSA age beyond 89 is capped at 90.

## Growth curve and shared parameter model

Growth curve or random effects model, assuming dropouts are missing at random, has been used with this sample when three waves were available (Zaninotto et al., 2009). Instead we used here the shared parameter model (Wu & Carroll, 1988) where the random effects influence both quality of life, 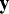 , and attrition, 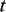 , such that given these, quality of life and attrition are independent. The likelihood is (Graham, Ryan, & Luszcz, 2011, p. 228):(1) 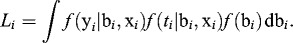



As in other joint models the underlying survival time is assumed normally distributed (Graham et al., 2011; Pantazis & Touloumi 2010; Touloumi, Babiker, Kenward, Pocock, & Darbyshire, 2003; Touloumi et al., 2003). Briefly, two models are jointly estimated, first, growth curve models with random effects, and second, attrition model where the random effects are included as covariate. Models are estimated in Latent Gold 4.5 (Vermunt & Magidson, 2006).

For comparison with the literature, we set out with analysis assuming missing at random attrition. Initially growth curve model is fit using covariates of age, random intercepts, random age rates, quadratic age and cohort dummies terms, assuming missing at random attrition. Then social status, health conditions and risk behaviours are entered block by block in models two to four. The final model allows the possibility of a comprehensive set of predictors (more than most studies) to contribute to explaining quality of life, enabling each predictor to have a net contribution (Fritzell & Lundberg 2007; Steptoe, Demakakos, & de Oliveira, 2012). Then the joint model is built using this comprehensive model as one part with the attrition model constituting the other part.

## Results

The sample at baseline is described in Table 1 where it appears that some predictors differentiate mean levels of CASP-19. There are more women in the sample, and they tend to report higher levels of quality of life. Along the quintile of wealth there is a gradient of life quality with the top quintile five points clear above the poorest quintile. Those with less than degree level education or those with routine occupation tend to have levels of life quality that are lower than those of their peers (with degrees or the intermediate and managerial classes). Older people diagnosed with health conditions have lower quality of life on average. These include cardiovascular conditions, chronic obstructive pulmonary diseases, and various forms of cancer. But older people engaging in physical exercise and abstaining from smoking show higher levels of quality of life.

**Table 1.** Description of baseline sample ELSA wave 1.

Covariate	Mean	SD	Covariate	Mean	SD
Age	62.1	8.1	Limiting illness		
Sex			None (68%)	45.3	7.1
Male (46%)	42.5	8.6	Yes (31%)	37.5	9.1
Female (53%)	43.1	8.6	CESD 8 items	1.43	1.9
Wealth quintile			ADL, IADL 0-11	0.45	1.2
1 (12%)	41.0	9.4	CVD		
2 (19%)	41.8	8.9	0 (52%)	44.0	8.0
3 (21%)	42.0	8.8	1 (47%)	41.6	9.0
4 (22%)	43.2	8.2	COPD		
5 (24%)	45.1	7.4	0 (93%)	43.2	8.4
Education			1 (6%)	37.9	9.7
<degree (87%)	42.5	8.7	Cancer		
Degree (12%)	45.3	7.3	0 (94%)	42.9	8.6
Cantril-like ladder	58.1	17.2	1 (5%)	41.6	8.9
Occupational class			Smoking		
Non-routine (59%)	44.1	8.0	Non-smoker (81%)	43.4	8.3
Routine (40%)	41.0	9.1	Smoker (18%)	40.5	9.6
Marital status			Drinking		
Single (5%)	41.8	8.7	No/light drinker (70%)	42.3	8.8
Married/cohabiting (73%)	43.4	8.3	Drink daily (29%)	44.3	8.0
Separate (21%)	41.2	9.3	Exercise: rigorous		
Soc. Support			No (79%)	42.2	8.8
0 (2%)	34.8	10.5	Yes (20%)	45.5	7.3
1 (98%)	43.0	8.5	Exercise: moderate		
Social connection (0-18)	9.8	3.9	No (38%)	40.3	9.4
			Yes (61%)	44.5	7.6

The bivariate associations between various determinants and quality of life need to be put into context since confounding is a real possibility, for instance routine workers (compared to managers) tend to accumulate less wealth during their working years. This calls for an exercise in longitudinal modelling of age trajectory.

**Figure 1.**
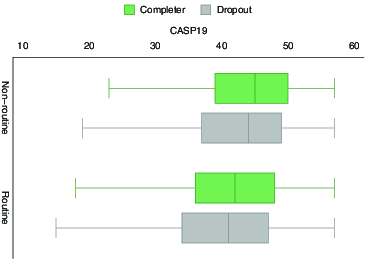
CASP-19, social class and dropping out.

But examining trajectories over time must proceed with care since the next illustration show how those who completed the longitudinal study until wave 5 (versus those who dropped out) are already different at baseline. Figure 1 shows this difference pattern in the case of social class: in addition to the pattern that older people with routine working past have lower quality of life as shown above, those who drop out also report lower quality of life. In fact in each occupational class the dropouts have lower life quality. This three-way association conveys a pattern where disadvantage is not merely associated with lower life quality but also with attrition. Thus assuming random attrition entails some risk.

## Trajectories of quality of life

Nonetheless we start with growth curve model of CASP-19 assuming missing at random as is common. Four models are estimated: initial model with sex and demographics, then with social stratification, followed by one with health, finally by one with behavioural risk factors of smoking, drinking and exercise. Then results from joint model follow.

The progression from the initial to the final model shows improvement in fit as evinced by lower BICs (Table 3). The more comprehensive the model is, the better the model becomes. The table presents annual rates of change in CASP-19 and level differences across many groups in society.

**Table 2.** Trajectories of CASP-19 in England 2002–2010.

	Initial	Social	Health	Behaviour	Joint
	Coef/SE	Coef/SE	Coef/SE	Coef/SE	Coef/SE
Sex, F	0.457†	1.155‡	1.283‡	1.349‡	1.438
	0.169	0.17	0.135	0.135	0.128
Depression era	−0.261	−0.313	−0.321	−0.31	−0.497
	0.803	0.721	0.58	0.575	0.581
Interwar cohort	−0.763	−1.057*	−0.711	−0.667	−0.744*
	0.507	0.458	0.372	0.369	0.364
War baby cohort	−0.337	−0.661*	−0.424	−0.397	−0.505*
	0.302	0.271	0.218	0.217	0.202
Age	1.594‡	1.637‡	1.180‡	1.114‡	1.112‡
	0.091	0.091	0.085	0.085	0.083
Age^2^	−0.013‡	−0.012‡	−0.009‡	−0.008‡	−0.008‡
	0.001	0.001	0.001	0.001	0.001
Married/coh.		0.941‡	0.625*	0.557*	0.540*
		0.321	0.264	0.262	0.246
Separated		−0.1	0.366	0.347	0.264
		0.33	0.274	0.272	0.255
Widowed		0.578	0.982‡	0.941	0.908
		0.348	0.292	0.29	0.277
Lower-middle Q		0.753	0.439	0.408	0.337
		0.286	0.226	0.224	0.219
Middle Q		1.191‡	0.578*	0.565*	0.517
		0.286	0.226	0.224	0.216
Upper-middle Q		1.911‡	1.093‡	1.013‡	0.911‡
		0.292	0.231	0.229	0.220
Top Q		3.096‡	1.769‡	1.614‡	1.452‡
		0.311	0.246	0.244	0.233
Degree		0.038	−0.316	−0.489*	−0.442*
		0.243	0.192	0.191	0.181
Routine workers		−1.191‡	−0.561‡	−0.458‡	−0.432‡
		0.167	0.133	0.132	0.128
Cantril-like ladder		0.102‡	0.093‡	0.092‡	0.094‡
		0.003	0.002	0.002	0.008
Social support		2.093‡	1.874	1.871	1.946
		0.29	0.275	0.274	0.271
Social connections		0.187	0.176	0.170	0.175
		0.011	0.01	0.01	0.010
Household size		0.033	−0.068	−0.074	−0.124
		0.089	0.071	0.071	0.064
Limiting illness			−2.249	−2.150	−2.171
			0.083	0.083	0.083
ADL IADL			−1.062	−1.016	−0.922
			0.034	0.034	0.033
CES-D			−1.141	−1.127	−1.150
			0.02	0.02	0.021
CVD			−0.183	−0.183	−0.158*
			0.069	0.069	0.069
COPD			−0.440	−0.418	−0.347
			0.133	0.133	0.134
Cancer			−0.415	−0.400*	−0.439
			0.161	0.161	0.160
Smoker				−0.666	−0.672
				0.130	0.126
Alcohol daily				0.378	0.358
				0.089	0.089
Vigorous exercise				0.627	0.615
				0.086	0.086
Mod exercise				0.623	0.540
				0.069	0.070
Constant	−6.501*	−21.869	−4.034	−2.245	−1.200
	3.259	3.26	2.959	2.956	2.883
Random int.					3.618
					9.598
Age					6.377
					0.064
Age					−0.648
					0.010
Female					0.053
					0.029
Constant					−15.201
					0.093
	6.519	5.350	4.060	4.071	0.001
	0.137	0.227	0.204	0.201	0.001
	058	0.058	0.041	0.040	0.073
	0.004	0.005	0.004	0.005	0.001
	19.961	19.750	18.824	18.783	19.046
	0.194	0.194	0.188	0.187	0.186
	0.20	0.20	0.44	0.45	0.61
BIC	199437	197103	192044	191877	305209
N	30654	30651	30651	30651	30651

Significance: * 5%, 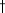 1%, 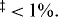

**Table 3.** *Top panel*: the peaks of the third age when attrition is assumed random in growth model versus when attrition in jointly modelled; *bottom panel*: the level-difference across cohorts in CASP-19, reference is the baby boomers cohort, based on the joint model.

Model	Peak age	Standard deviation	Upper peak
Growth curve	66.4	5.8	77.7
Joint model	67.0	5.8	78.3
	Difference in	Standard	Max
Cohort	CASP-19	deviation	difference
War baby	−0.51	0.20	−0.90
Interwar	−0.74	0.36	−1.46

Initially the annual rate of change and the acceleration of change are 1.594 and −0.013; both are significant (*p* < 0.001). No longitudinal study has found both to be significant. These estimates suggest that beyond midlife a sustained improvement in life quality is a possibility and in fact the peak is reached at about 63 years of age. By the final model the peak is estimated to happen at about 66. The progression across the models shows not only better fit but also reveals later timing of peak quality of life. The more comprehensive the model is, the further the onset of decline is pushed back.

There is no difference across the cohorts born during the Depression era, born between the wars, and born during the war compared to the early baby boomer cohort. But there are differences across social groups. Those married or cohabiting have higher quality of life compared to singles (the reference). Consistent with the bivariate association wealth confers higher life quality with the top three quintiles particularly and significantly higher than the rest. A notable exception is degree where in the final model it is negatively associated with life quality. This is known in Britain (Clark & Oswald, 1994) and in the US (Kahneman & Deaton, 2010). But like most social stratification, social class indicators both objective (NS-SEC) and subjective (Cantril ladder) are associated with life quality in the expected direction. Older people with routine working past (or present) or who perceived to be near the bottom of the ladder tend to have lower life quality.

Often arising in discourse on ageing are social support and social connections cf. Zaninotto et al. (2009). These are important and statistically significant. In all models, they are stable and sizable, for instance in the final model social connections (range 0–18) have an estimate of 0.170.

The penultimate and final models differ in that one has health conditions while the other has health behaviours in addition. The coefficients for health conditions in the two models are all significant, comparable and intuitively signed. Thus these reduce quality of life: depression, cardiovascular conditions, chronic obstructive pulmonary diseases, cancer or malignant tumours, as well as problems doing daily activities or instrumental activities.

All the behavioural risk factors are effective with smoking being particularly harmful as it is estimated to reduce life quality by 0.666 (*p* < 0.001). Having a drink on a daily basis is shown to have a positive association with life quality (consistent with (Steptoe, Shankar, Demakakos, & Wardle, 2013)) but not as large as doing moderate to vigorous physical exercise which has an estimate of 0.623 (*p* < 0.001).

Remarkably the joint model now reveals cohort coefficients to be statistically significant and quantitatively important. The difference between the most recent cohort and the interwar cohort is −0.744 ( *p* = 0.041). Compared to the estimates from the final growth curve model assuming attrition at random, the joint estimates for most predictors are stable and if anything somewhat stronger. The simple survival part suggests predictors including age vary significantly with hazard of dropping out. This gives some confidence about the interpretation to put on the joint model results.

**Figure 2.**
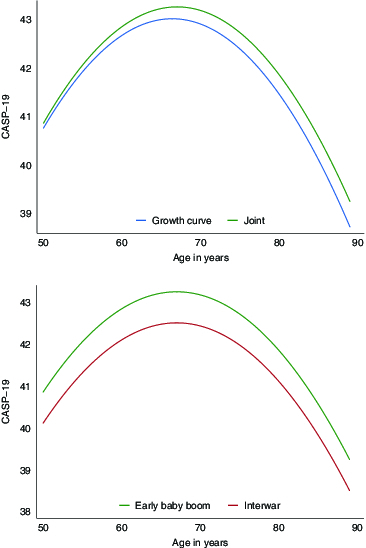
Comparing trajectories of cohorts. *Top panel*: joint model (higher overall) accounts for attrition while growth curve model assumes attrition at random; *bottom panel*: both models account for attrition and show early baby boomers have higher CASP-19 scores than those born between the wars.

To highlight the comparison between ignoring and accounting for attrition and what it does to cohort differences, Figure 2 shows the curvilinear trajectories of quality of life in later life drawn using the significant coefficients of age and age-squared in Table 2. The top panel compares the joint model where attrition is accounted for versus the growth curve model where attrition is assumed random (for the reference cohort that is the early baby boomer); the bottom panel compares the reference cohort versus the interwar cohort in the joint model. To focus on the difference, the origin is arbitrarily set at the 25th percentile of CASP-19. This does not affect the difference shown between the trajectories; were the origin set at the 52nd percentile, the following discussion will still hold. Note that the green trajectories (higher ones) in both panels refer to the same cohort and same model, i.e. early baby boomer in joint model. The top panel shows that the joint model manages to recover curvilinear trajectory of CASP-19 that is more encouraging or higher than when attrition is ignored. Moreover, when one settles on the joint model (bottom panel), secular improvement experienced by recent cohort compared to earlier cohorts appears.

## Discussion

The joint model makes two major contributions and a few notable ones. First, unlike models ignoring attrition, it uncovers secular change in quality of life affecting the lives of people born to different cohorts. The early baby boomers attain higher quality of life compared to their older peers, the war baby and interwar baby cohorts. The magnitude of this improvement as visible throughout the later life illustrates the difference in life quality when reaching older ages at the turn of the century versus at the end of the last one.

It is worth remarking that this is the first evidence of secular, i.e. cohort-based improvement in the levels of life quality in older ages based on nationally representative sample. To what can this improvement be attributed to? In his discussion of secular improvement in cognition in older ages, or the Flynn effect related to the ageing brain, Whalley (2001, p. 56ff) highlighted several institutional, environmental and societal changes. They include education that has been increasingly and widely available and that is better structured to deliver chunks of learning suitable to different learning abilities. Importantly learning has been increasingly broadened to include not only mastery of facts but also cultivation of autonomy and self-realisation as in the common phrase ‘independent learning’. Autonomy and self-realisation are of course at the heart of CASP-19.

Nutrition is another factor that has improved over the last century. Our finding that physical activities bear on quality of life is congruent with this. Recent cohorts are better nourished and are physically more able to be active and thus improve their life quality.

Public health is also another major advance over the lives of these different cohorts. Both in its preventive and curative arms, public health initiatives have led to marked improvement in people's general health in Britain and many countries (Rose, 1992/2008; Deaton, 2013). Although our analysis includes large number of health conditions, more than in previous investigations, this list is not claimed to be complete. So general improvement in public health across cohorts may be partially responsible for the secular improvement in life quality.

Second, the analysis reveals non-linear change in age-dependence trajectory that reaches its peak at about 67. This contrasts with previous longitudinal which finds no peak when health conditions are considered (Zaninotto et al., 2009). In their final analysis the trajectories are practically linear as the age square coefficient is no different from zero. This is at variance with findings from cross-sectional study of Netuveli et al. (2006). Now if one takes the cross-sectional linear and square age effects from their study, the peak is calculated to be at 64 years. This is somewhat earlier than what is found here. Unfortunately no significance can be calculated for their case since estimated variance-covariance matrix is not reported.

But our study also has limitations. CASP-19 is one important measure that has allowed us to see much change and multiple inequalities: level changes across cohorts and curvilinear changes across years as well as inequalities between groups in society. But CASP-19 is only one measure. Other measures of quality of well-being should also be looked at including those that are broader or less specific in conception such as life evaluation and happiness (Fleurbaey, 2009; Fleurbaey & Blanchet, 2013; Layard, 2005; Pierewan & Tampubolon, 2013, 2014a, 2014b). Our exploration in this study also stopped at level change across cohort, postponing interactions with age and other variables to further work. This is due to the large set of predictors already considered and to the fact that level change across cohort has not been demonstrated before.

## The peak of the third age

This analysis found that longitudinal trajectories are curvilinear in form. Unlike previously (Zaninotto et al., 2009), we demonstrated that beyond the age of 50 quality of life does not relentlessly march to the tune of decline. Given this form, it is possible to give a more precise object to the contention or intuition that life quality will reach a peak and start to decline, soon entering the fourth age of Laslett. To give a sharper delineation to the trajectories in the third age, we compute peak ages for various models with their upper peak or limit (2.5%) and the implied standard deviation computed using Fieller's theorem (Fieller, 1932, 1954; Hirschberg & Lye, 2010); these are shown in Table 2 top panel. It shows that the joint model uncovers marked improvement in quality of life both in its timing and its extent. Instead of peaking at the age of 66.4, when attrition is taken into account more than six months further is expected. For the top 2.5% of the population peak age will not be reached until the age of 78.3.

Also included in the bottom panel are the cohort differences in the levels of quality of life revealed by the joint model, with the early baby boomer as the reference. Secular improvement in life quality has evidently been enjoyed by the more recent cohorts with a difference of three-quarters and up to one and a half points.

As can be expected from a measure of life quality that is separate from its predictors, CASP-19 allows comprehensive predictors including wealth, social and economic status, health conditions and behaviours to be included. This inclusion has yielded results that are enriching our understanding of older people life quality, commending the measure for further use. The results show that not only health risk behaviours such as smoking are damaging to health, they are also damaging to quality of life beyond their effects on health; similarly with salutogenic practice such as vigorous physical exercise which is beneficial to both health and quality of life. In addition, health conditions ranging from problems with daily activities to cancer all have negative associations with life quality in older people.

One puzzle is raised by educational attainment; specifically, those with degree education tend to present lower levels of life quality. This negative effect on well-being is known on both sides of the Atlantic (Clark & Oswald, 1994; Kahneman & Deaton, 2010). This again distinguishes CASP-19 from health, which invariably relates positively with higher levels of education.

Other social determinants of health can be expected to also affect life quality as this is an umbrella concept incorporating control, autonomy, realisation of self and pleasure. These have direct connections with social factors such as social connections and social capital (Helliwell & Putnam, 2004; Kawachi & Berkman, 2000; Tampubolon, 2012; Tampubolon, Subramanian, & Kawachi, 2013). Social connections through email, telephone or face-to-face meeting are not merely instrumental in exchanging useful information for maintaining health and well-being, they can also be reaffirming of one's sense of self and be a pleasure in themselves. Policy for healthy ageing that seriously considers social connections or social capital could yield benefits beyond good health into the sense of control, autonomy, pleasure and self-affirmation.

In conclusion, worthwhile societal aims are often jointly pursued: removing predicament and enabling attainment. Removing child poverty is one example. Perhaps stretching peak age is another. At the moment only one in 40 achieves peak quality at the age of at least 78. Society must consider whether it is desirable that one in five must remain at the peak quality of life by the age of 85 say. With CASP-19 and joint model at hand to give evidence, this is one debate worth having.
